# Obesity mechanism after hypothalamic damage: Cohort analysis of neuroimaging, psychological, cognitive, and clinical phenotyping data

**DOI:** 10.3389/fendo.2023.1114409

**Published:** 2023-03-28

**Authors:** Miwoo Lee, Min-Jung Park, Kyung Hwa Lee, Jung Hee Kim, Hyung Jin Choi, Yong Hwy Kim

**Affiliations:** ^1^ Department of Biomedical Sciences, Seoul National University College of Medicine, Seoul, Republic of Korea; ^2^ Department of Neurosurgery, Seoul National University College of Medicine, Seoul, Republic of Korea; ^3^ Division of Child and Adolescent Psychiatry, Department of Psychiatry, Seoul National University Hospital, Seoul, Republic of Korea; ^4^ Pituitary Center, Seoul National University College of Medicine, Seoul, Republic of Korea; ^5^ Department of Internal Medicine, Seoul National University College of Medicine, Seoul, Republic of Korea

**Keywords:** hypothalamus, fMRI, attention, craniopharyngioma, cognitive task, hypothalamic obesity

## Abstract

**Objective:**

The hypothalamus regulates energy homeostasis, and its damage results in severe obesity. We aimed to investigate the multifaceted characteristics of hypothalamic obesity.

**Methods:**

We performed multidimensional analyses of brain structure/function and psychological and behavioral phenotypes in 29 patients with hypothalamic damage (HD) (craniopharyngioma) and 31 controls (non-functional pituitary adenoma). Patients underwent structural and functional magnetic resonance imaging and completed self-reports and cognitive tasks.

**Results:**

Patients with HD showed significantly higher postoperative weight gain than controls. The HD group also showed significant hypothalamic damage and lower neural activation in the left caudate nucleus in response to food images. The HD group had significantly higher food inattention, lower satiety, and higher restrained eating behavior. Within the HD group, higher restrained eating behavior was significantly associated with lower activation in the bilateral fusiform gyrus.

**Conclusion:**

These results suggest that hypothalamic damage contributes to weight gain by altering the brain response, attention, satiety, and eating behaviors. The present study proposes novel neuro-psycho-behavioral mechanisms targeted for patients with hypothalamic obesity.

## Introduction

The hypothalamus is the primary site for the regulation of energy homeostasis. Given the critical function of the hypothalamus, damage to the hypothalamus may lead to several detrimental effects. For example, hypothalamic obesity is a form of obesity caused by structural damage in the hypothalamus (1–3). Hypothalamic obesity occurs in approximately half of the patients who have undergone surgical removal of tumors near the hypothalamus, such as craniopharyngioma. Hypothalamic obesity is the most concerning complication and is often developed despite the adequate replacement of pituitary hormone deficiencies (3, 4). Moreover, it is associated with severe sequelae and negatively impacts the quality of life (5). However, there is a lack of evidence regarding definitive diagnostic guidelines or mechanisms to evaluate the extent of hypothalamic damage and the subsequent psychological and behavioral dysfunctions (2). Therefore, studies on the features of hypothalamic obesity based on multimodal aspects are required, which may consequently contribute to clarifying diagnostic guidelines and developing interventions for hypothalamic obesity.

Previous studies have shown that hypothalamic obesity is associated with abnormal eating behavior and psychologies (6–8). For example, pathological eating behaviors (e.g., higher eating restraint scores, snacking, night eating, and altered attitudes toward food) have been reported among patients with hypothalamic obesity (7, 9, 10). Psychological abnormalities (e.g., sleepiness, mood, and personality) have been reported to be the leading cause of abnormal eating behaviors (10). However, these studies were conducted to examine pathological eating behaviors and psychological characteristics independently. This lack of integration between behavioral and psychological characteristics may limit the comprehensive and integrated understanding of hypothalamic obesity. The present study attempted to elucidate both the psychological and behavioral characteristics of patients with hypothalamic obesity.

Furthermore, the direct mechanism linking hypothalamic damage to abnormal eating behavior is thought to involve neural circuit malfunctions related to eating (5, 11). This suggests that examinations of neural function as well as psychological/behavioral assessments in patients with hypothalamic obesity may be required. Indeed, a few functional magnetic resonance imaging (fMRI) studies have investigated altered brain activation in patients with hypothalamic obesity (12, 13). However, these studies did not focus on food or obesity-related fMRI mechanisms of hypothalamic obesity through multiple-dimension analyses, including hypothalamic structural damage, whole-brain response to food, food-related cognition, food-related psychology, eating behaviors, and weight gain. We hypothesized that hypothalamic damage could alter brain functions for satiety, food attention, and inhibitory control, which could result in unhealthy behavior and weight gain.

## Methods

### Participants

This study included 29 patients and 31 controls who underwent surgeries for craniopharyngioma (hypothalamic damage group) and non-functional pituitary adenoma (control group), respectively (pair-matched based on age, gender, preoperative BMI, and hormone function status). The definition of “the HD patient” of the present study was the patient who had potential hypothalamic functional damage due to surgery for craniopharyngioma. The exclusion criteria were as follows: age <19 years, on medications that could significantly influence weight gain (besides hormone therapy), psychiatric problems, severe postoperative complications (bleeding, infection, or pituitary apoplexy), metastasis, previous treatment history (surgery, gamma knife, or radiation therapy), and visual disturbances. This study was reviewed and approved by the Institutional Review Board of SNUH (IRB No.1710-096-895).

### Procedures

**Figure 1:**
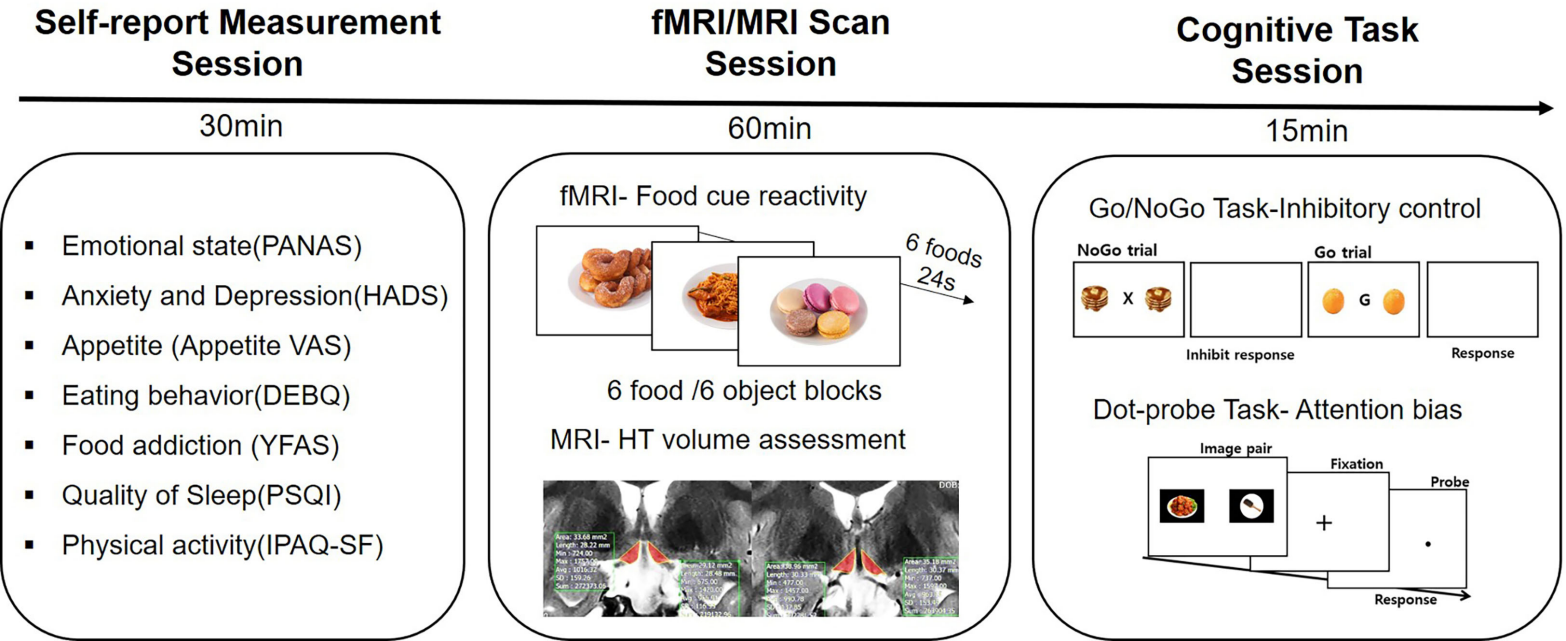
Study design. All participants filled out self-reported questionnaires for assessing psychological and behavioral phenotypes. They underwent an fMRI scan and a routine follow-up MRI scan. After the MRI scan, two computerized cognitive tasks were performed.

Patients were asked to arrive at the MRI center an hour before the MRI scan began. First, patients were asked to complete nine Korean standardized questionnaires. Then, patients underwent a routine follow-up structure MRI sequence. This was followed by an fMRI scan during the food image presentation. After finishing the MRI/fMRI scan, they were instructed to complete the Go/NoGo and dot-probe tasks (**Figure 1**).

### Psychological measurements

We used the following self-reported measurements for the psychological and behavioral assessments: Appetite visual analog scale (VAS) (14) for the appetite and internal state, Positive and Negative Affect Scale (PANAS) (15) for the current affect state, Hospital Anxiety and Depression Scale (HADS) (16) for pathological mood disorders, Dutch Eating Behavior Questionnaire (DEBQ) (17) and Yale Food Addiction Scale (YFAS) (18) for eating behavior, and International Physical Activity Questionnaire Short Form (IPAQ-SF) (19) and Pittsburgh Sleep Quality Index (PSQI) (20) for other eating-associated behaviors.

### Individually tailored hypothalamus volumetric method

We assessed the volume of the damaged hypothalamus (absolute volume of the hypothalamus) on a T2-weighted MR image [slice number = 15, voxel size = 0.4 × 0.5 × 3 mm^3^, matrix = 320 × 256, slice thickness = 3 mm, repetition time (TR) = 3,000 ms, echo time = 127.2 ms, flip angle = 160]. A well-trained neuroimaging analyst manually segmented the hypothalamus area using the freehand option of the SNUH PACS program (INFINITT Co. Ltd., South Korea), based on a method recently developed by our team (21). We modified the lateral border of the optic tract to adjust the thalamus boundary for each patient in T2-weighted MR images to exclude thalamus areas from hypothalamic volume measurements [method 2 in ref (21)]. The intracranial volume was calculated from the segmentation function of the magnetization-prepared rapid gradient echo (MPRAGE) sequence using the CAT12 (a Computational Anatomy Toolbox for SPM) (22) for whole-brain correction. To adjust for individual head size differences, we calculated the relative hypothalamus volume by dividing the hypothalamus volume by the intracranial volume.

### Functional MRI

#### Image acquisition

We collected the imaging data on a 3.0-T scanner (SIEMENS MAGNETOM TrioTim syngo MR B17, Germany) using a 32-channel sensitivity encoding head coil. We used an MPRAGE sequence (192 contiguous sagittal slices with TR = 2,100 ms, echo time = 3.74 ms, flip angle = 10, voxel size = 0.9 × 0.9 × 1 mm^3^, and matrix = 256 × 256) to obtain a high-resolution 3-D volume image. fMRI data were obtained (198 volumes with TR = 2,000 ms) with T2-weighted single-shot echo planar imaging sequences. Each participant was axially scanned using the following parameters: voxel size = 3.4 × 3.4 × 3.4 mm^3^, slice number = 38 (interleaved), matrix = 80 × 80, slice thickness = 3.4 mm, slice gap = 0 mm, TR = 2,000 ms, TE = 30 ms, and FOV = 240 × 240.

#### Food image perception task

We used pictures of palatable food stimuli (23) and non-food object stimuli for the in-scanner visual perception task (45 images each). We presented the task on a projector screen using the E-Prime software (Psychology Software Tools, Pittsburgh, PA, USA). The palatable food images and non-food object images were presented in a block design format during the two experimental runs. The two runs were counterbalanced. Each run consisted of six (food blocks and object blocks, three each) blocks comprising 24 s. Six individual images for 4 s from the list of 15 stimuli were randomly presented in each block. Each block was separated by 8 s. Moreover, each run began with a blank screen comprising a fixation cross for 16 s.

#### fMRI data analyses

We preprocessed and analyzed the whole-brain functional images using the SPM12 software package (Wellcome Department of Imaging Neuroscience, London, United Kingdom). They were run in MATLAB 9.4.0 (R2018a, The MathWorks Inc., Natick, MA, USA). The functional images were slice time-corrected, realigned, and co-registered. They were then spatially normalized to the standard Montreal Neurological Institute brain template. These images were smoothened using an isotropic Gaussian kernel with a 6-mm complete width at half maximum. Moreover, we used Artifact Detection Tools (http://www.nitrc.org/projects/artifact_detect) for detecting the outliers. Single scans identified as invalid outliers were removed from the subsequent analysis.

Subject-level analyses included the following two regressors per model: visual exposure to food images and object images. Head motion parameters and outliers were also included in the model as covariates to control the effects of motion and outliers. For each subject, we calculated the following contrast image for each subject: food *vs*. object. Group-level analyses were conducted using two-sample *t*-tests to compare group differences in neural activation in response to food images (*vs*. object images). We reported on the fMRI results with a significance level of *P* = 0.005 (uncorrected) and a cluster extent threshold of *k* = 30 contiguous voxels, as recommended by previous studies (24, 25). Food-related regions of interest (ROIs) included the bilateral amygdala, caudate, putamen, fusiform, insula, nucleus accumbens, and orbitofrontal cortex (26–28). We defined the ROIs using Automated Anatomical Labeling 3. However, the ROI for the nucleus accumbens was defined by the Harvard-Oxford Subcortical Structural Atlas. The MarsBaR toolbox (http://marsbar.sourceforge.net/) was run in Matlab 9.4.0 (R2018a; The MathWorks Inc., Natick, MA, USA) to extract the mean beta values from the clusters, showing significant group differences from the whole-brain *t*-tests.

### Computerized cognitive tasks

All computerized cognitive tasks were prepared using PsychoPy3.0 and run on a 15-inch Dell laptop. All food image stimuli used in the tasks were selected from the food image set for Koreans (23).

#### Go/NoGo task

We implemented the Go/NoGo task protocol postulated by Jasinska et al. (29), which is modified to include flanker food distractors, which eventually facilitated an assessment of the efficiency of inhibitory control in the presence of food images. We included 90 palatable food images for the distracters. The Go/NoGo task measures the efficiency of response inhibition or inhibitory control (30), as indexed by the number of false alarms on NoGo trials (i.e., commission error rate). A higher rate of false alarms indicates a greater deficit in inhibitory control. We measured the lapse of attention to cue by the number of no responses in the Go trials (omission error rate). A higher rate of error in the Go trial indicated a greater deficit in attention. Reaction time indicates response/attention bias. We included food distracter trials to maximize the number of false alarms committed per category. The participants observed a target letter in the middle of the screen, flanked by two identical food distracter images. We instructed them to press the space bar for all letters except the letter X (Go trials, 66% of all trials) and to inhibit their response for the letter X (NoGo trials, 33% of all trials). The Go targets included the letters G, Q, and O. Each trial consisted of a target stimulus and two flanker food distractors presented for 500 ms, followed by a white screen presented for 1,000 ms. The total response limit was 1,500 ms. After the instructions and following eight practice trials, the participants completed three runs of tasks each consisting of 90 trials (60 Go trials and 30 NoGo trials), for a total of 270 trials (180 Go trials and 90 NoGo trials, respectively). The instructions emphasized on both speed and accuracy. The order of trials was randomized across the three blocks for each participant. All participants were provided with the same food distracter images.

#### Dot-probe task

We employed a food visual image-specific dot-probe task to assess the attentional bias to food. We obtained 104 and 56 food and non-food object pictures, respectively, for the stimuli. There were 168 experimental trials that comprised the following in picture pairs from each category: 56 food/object (congruent trial), 56 food/object (incongruent trial), 28 food/food (neutral), and 28 object/object (neutral). Their order of presentation was randomized for each participant, with a restriction on the immediate repetition of the preceding pair. The fixation was presented for 500 ms in each trial, followed by a separation of the pairs for 1,000 ms. Moreover, one picture each was presented left and right to the central point. Immediately following an offset of the picture pair, a small dot appeared in place of one of the two pictures, in line with previous research using the dot-probe procedure (31–34). Each picture within the pair was likely equal to fall in either the left or the right position on the computer screen. Furthermore, the dot was equally likely to replace either the food (congruent trial) or the object (incongruent trial) picture. Differences in the reaction time of each type of trial are indexed as the attention bias, initial attentional orientation (i.e., attention orienting), and disengagement of attention from the visual cue (i.e., attention disengagement) (31, 35). The participants were instructed to fix their eyes on the cross at the center of the screen every time it appeared. Furthermore, we asked them to rapidly and accurately indicate the point where the dot appeared by pressing either a left or right response key on the keyboard. The computer recorded the response time to the nearest millisecond and the number of errors. The participants were provided a break for 30 s, halfway through the trial.

### Statistical analyses

We analyzed the psychological, behavioral, and anthropometric data using the SPSS Statistics software (version 25, Armonk, NY, USA). The *P*-values are from an unpaired, two-sample *t*-test that compared patients in the HD group to those in the control group. We conducted independent sample *t*-tests to compare the percentage of damaged hypothalamus volume and the mean volume of each group. The *t*-value represents the difference between the sample mean and the population mean, standardized by the standard deviation of the sample. The correlation between all anthropometric, psychological, and behavioral data and the ROI beta values was assessed using the Pearson product-moment correlation coefficient (two-tailed, *P* < 0.05).

## Results

### Demographic and clinical characteristics

Sixty-one participants completed the entire study procedure. Two patients failed to complete the fMRI scan due to a problem with the fMRI projector. We successfully collected the fMRI data of 55 of the 59 eligible patients (93%), and the data of four patients were excluded due to a technical issue of data transfer (*N* = 3) and poor vision (*N* = 1). We excluded the data of two patients in the Go/NoGo task [one outlier (error rate > 30%) and one missing data].

The demographic and clinical characteristics of the study participants are shown in **Table 1**. There were no differences in age, preoperative weight, and preoperative BMI between the hypothalamic damage (HD) group (craniopharyngioma, *n* = 29) and the control group (non-functional pituitary adenoma, *n* = 31). There was a significant difference in the Mueller grading (36) and follow-up duration (the period between surgery and participation in this study).

**Table 1:** Demographic and clinical characteristics.

	Hypothalamic damage (*n* = 29)		Control (*n* = 31)	*P*-value
Age^a^	48.00 ± 12.77		48.77 ± 13.10	0.818
Sex, male, *n* (%)	16 (55.2)		16 (51.6)	0.782
Follow-up duration (month)^b^	40.67 ± 25.87		23.16 ± 20.18	0.005*
Preoperative body weight	67.54 ± 12.70		70.5 ± 13.30	0.383
Preoperative BMI	25.06 ± 3.24		25.23 ± 3.14	0.836
Postoperative BMI^c^	26.88 ± 4.32		25.38 ± 3.54	0.145
Mueller grading, *n* (%)				
0	5 (17.2)		25 (80.6)	0.000*
1	9 (31.0)		5 (16.1)	
2	15 (51.7)		1 (3.2)	

Data are expressed as mean ± SD or n (%). Hypothalamic damage (HD) group: craniopharyngioma; control group: non-functioning pituitary adenoma. The P-value presented was related to the unpaired, two-sample Student’s t-test comparing the hypothalamic damage group with the control group. The chi-square P-value presented for sex and Mueller grading.

a Age when our patients participated in this study.

b Period between surgery and participation in this study.

c BMI when our patients participated in this study.

* indicates significance (P<0.05).

### Weight change

**Figure 2:**
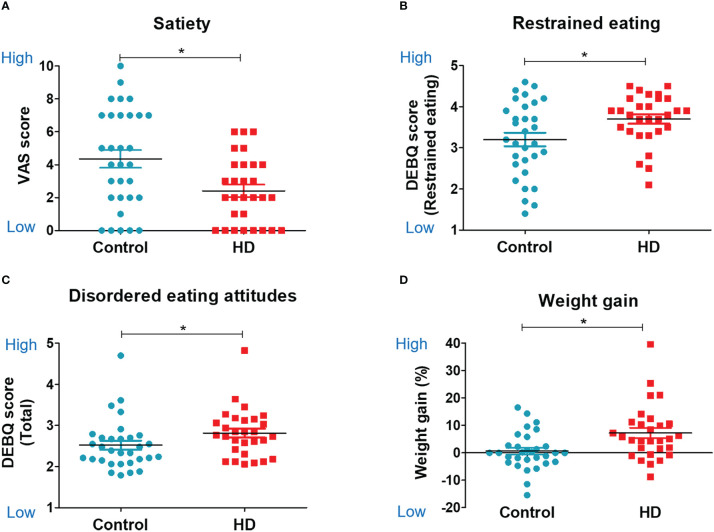
Food-related psychology, eating behavior, and weight change. Scatter plots for food-related psychology, behavior, and weight change in the hypothalamic damage (HD) and control groups. The unpaired, two-sample Student’s *t*-test was used for comparing the HD and control groups. **(A)** Visual analog scale score of satiety. **(B)** Restrained eating subscale of DEBQ (Dutch Eating Behavior Questionnaire). **(C)** Total score of DEBQ. **(D)** Weight gain %. * indicates significance (P<0.05).

Patients in the HD group gained greater postoperative weight compared with the control group. There was a 7.23% increase in postoperative weight in the HD group, while only a 0.63% increase was observed in the control group (*t* (58) = 3.02, *P* = 0.004, **Figure 2D**). A total of 51.72% of patients with HD (15 of 29) had a weight gain greater than 5%, and 31.03% of patients with HD (9 of 29) had a weight gain greater than 10% after the surgery.

### Structural MRI: The degree of hypothalamic damage

**Figure 3:**
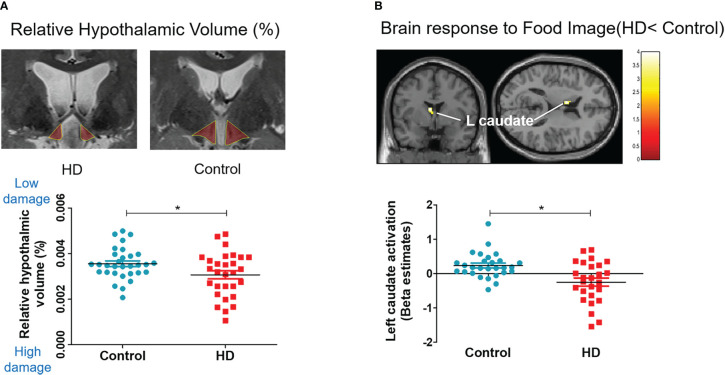
Hypothalamic damage and whole-brain response to food. Scatter plots for relative hypothalamic volume and brain response difference in the HD and control groups. The unpaired, two-sample Student’s *t*-test was used for comparing the HD and control groups. **(A)** Relative volume: hypothalamic volume percentage; (Hypothalamic volume/Total intracranial volume) * 100. **(B)** Left caudate activation to food image (food > object contrast).

The HD group showed a significantly lower absolute volume of the hypothalamus (667.63 *vs*. 782.54, *t* (58) = −2.351, *P* < 0.05) and relative volume of the hypothalamus (hypothalamic volume percentage; [hypothalamus volume/total intracranial volume] * 100), indicating significant hypothalamic damage, compared with the control (0.0460 *vs*. 0.0534, *t* (58) = −2.017, *P* < 0.05, **Table 2**; **Figure 3A**).

**Table 2:** Neuroimaging, psychological, cognitive, and clinical phenotyping of the hypothalamic damage group.

Dimension	Phenotype	Variables	Hypothalamic damage (*n* = 29)	Control (*n* = 31)	*P*-value
Hypothalamic structure	Hypothalamic damage	Absolute volume of the hypothalamus (mm^3^)	667.63 ± 224.50	782.60 ± 148.92	0.022*
		Relative volume of the hypothalamus (%)	0.0460 ± 0.01637	0.0534 ± 0.01085	0.043*
Food-related cognition	Go/NoGo task				
	Inattention	Go RT (s)	0.50 ± 0.07	0.47 ± 0.06	0.075
	Attention	Go errors (%)	9.13 ± 8.11	4.09 ± 4.19	0.005*
	Inattention	NoGo errors (%)	8.02 ± 6.83	6.30 ± 4.93	0.274
	Dot-probe task				
	Attention bias	RT difference (IT − CT) (ms)	−1.04 ± 18.39	3.00 ± 21.42	0.437
	Attention orienting	RT difference (N − CT) (ms)	−1.64 ± 20.20	−0.03 ± 15.99	0.733
	Attention disengagement	RT difference (IT − N) (ms)	0.60 ± 15.40	3.04 ± 25.18	0.655
Food-related psychology	Hunger	Visual analog scale	2.66 ± 2.59	2.68 ± 2.84	0.975
	Satiety	Visual analog scale	2.41 ± 2.03	4.35 ± 3.01	0.005*
	Fullness	Visual analog scale	2.97 ± 2.47	4.35 ± 3.01	0.303
	Prospective to eat	Visual analog scale	4.62 ± 2.01	4.26 ± 2.34	0.523
	Tasty	Visual analog scale	1.76 ± 1.49	1.70 ± 1.77	0.888
	Craving	Visual analog scale	2.52 ± 2.28	2.84 ± 2.85	0.633
Eating behavior	Disordered eating attitudes	DEBQ (total)	2.81 ± 0.58	2.52 ± 0.61	0.064
	Restrained eating	DEBQ_R	3.7 ± 0.6	3.2 ± 0.91	0.016*
	Emotional eating	DEBQ_Emo	1.98 ± 0.92	1.62 ± 0.86	0.137
	External eating	DEBQ_Ext	3.01 ± 0.71	3 ± 0.68	0.969
	Food addiction	YFAS	1.41 ± 1.24	1.29 ± 1.47	0.727
Weight change	Weight (%)	Body weight change (%)	7.24 ± 9.98	0.63 ± 6.77	0.004*
	Weight (kg)	Body weight change (kg)	5.31± 7.90	0.85 ± 4.66	0.006*
	BMI	BMI change (%)	7.2 ± 9.99	0.58 ± 6.76	0.004*
Mental and physical health	Emotion	PANAS	0.56 ± 0.10	0.56 ± 0.10	0.848
	Anxiety	HADS anxiety	5.03 ± 2.82	6.06 ± 2.61	0.147
	Depression	HADS depression	7.31 ± 3.71	6.81 ± 2.88	0.558
	Sleep quality	PSQI (total)	8.07 ± 3.40	7.58 ± 3.88	0.607
	Physical activity	IPAQ-sf (total)	4,673.85 ± 6,773.40	3,585.70 ± 4,567.46	0.492

Data are expressed as mean ± SD or n (%).

RT, reaction time; IT, incongruent trial; CT, congruent trial; N, neutral; DEBQ, Dutch Eating Behavior Questionnaire; DEBQ_R, DEBQ Restrained Eating; DEBQ_Emo, DEBQ Emotional Eating; DEBQ_Ext, DEBQ External Eating; YFAS, Yale Food Addiction Scale; PANAS, Positive and Negative Affect Schedule; HADS, Hospital Anxiety and Depression Scale; PSQI, Pittsburgh Sleep Quality Index; IPAQ-sf, Physical Activity Questionnaire (IPAQ) - Short Form. * indicates significance (P<0.05).

### Functional MRI: Brain response to food

The whole-brain analysis revealed that the HD group showed significantly lower activation in the left caudate nucleus, which is associated with reward, emotion, and motivation function (**Figure 3B**) when they viewed food images (*vs*. object images). The HD group also showed significantly higher activation in the right occipital gyrus and inferior frontal gyrus in response to food images (*vs*. object images) compared with the control group (**Supplementary Table 1**).

### Computerized tasks: Food-related cognition

**Figure 4:**
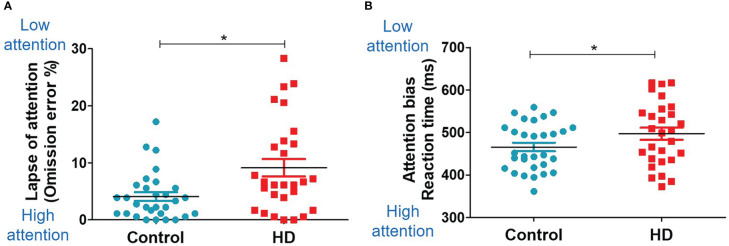
Food-related cognition (Go/NoGo task). Scatter plots for the Go/NoGo task results in the HD and control groups. The unpaired, two-sample Student’s *t*-test was used for comparing the HD and control groups. **(A)** Group difference in the omission error rate (missing response to the Go trial). **(B)** Group difference in the reaction time. * indicates significance (P<0.05).

The HD group showed significantly higher food inattention (lapses of attention, omission error %; not responding to the go cue in the Go/NoGo task) than the control group (9.13 ± 8.11 *vs*. 4.09 ± 4.19, *t* (56) = 3.00, *P* = 0.004, **Figure 4A**). The HD group also showed a trend of higher food inattention (attention bias, reaction time to the go cue in the Go/NoGo task) than the control group (0.50 ± 0.07 *vs*. 0.47 ± 0.06, *t* (56) = 1.86, *P* = 0.075, **Figure 4B**). No significant group differences were observed in the performance of the dot-probe task.

### Self-report assessments: Food-related psychological characteristics

The HD group showed a lower satiety VAS score than the control group (2.41 ± 2.03 *vs*. 4.35 ± 3.01, *t* (58) = −2.91, *P* = 0.005, **Figure 2A**). There were no significant group differences in other psychological and cognitive measurements (**Table 2**). The HD group showed higher restrained eating behavior (restrained eating score from the DEBQ) than the control group (3.7 ± 0.6 *vs*. 3.2 ± 0.91, *t* (58) = 2.47, *P* = 0.02, **Figure 2B**). The HD group also showed a trend of higher unhealthy eating behavior (total score from the DEBQ) than the control group (2.81 ± 0.58 *vs*. 2.52 ± 0.61, *t* (58) = 1.89, *P* = 0.06, **Figure 2C**). There were no significant group differences in other psychological and cognitive measurements (**Table 2**).

### Correlation analysis within the HD group

**Figure 5:**
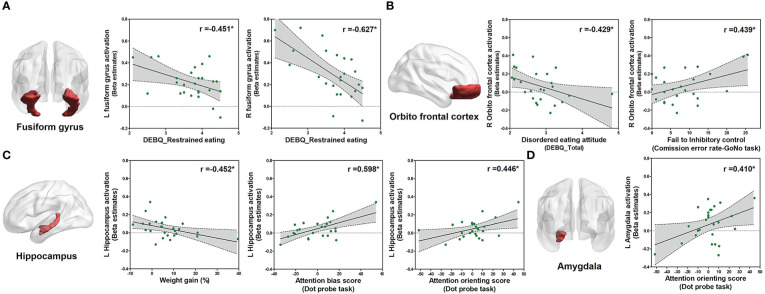
Correlation analysis within the HD group. Correlations between eating behavior and the parameter of estimated beta value of ROIs within patients with HD (*n* = 29) using Pearson product-moment correlation coefficient (two-tailed, *P* < 0.05). The *Y*-axis means the activation level of each ROI in food > object contrast. **(A)** Correlation between the restrained eating score and left/right fusiform gyrus activation. **(B)** Correlation between the disordered eating attitude (DEBQ total score) and inhibitory control with activation of the right OFC. **(C)** Correlation between the weight gain and attention scores from the dot-probe scores with left hippocampus activation. **(D)** Correlation between attention and activation of the left amygdala.

Since restrained eating was the major abnormal behavior observed in the HD group, further correlation analysis was performed within the HD group. Interestingly, higher restrained eating was significantly associated with lower activation in the left (*r* = −0.451, *P* = 0.021, **Figure 5A**) and right (*r* = −0.627, *P* = 0.001, **Figure 5A**) fusiform gyrus. Additionally, a higher disordered eating attitude was significantly associated with lower activation of the right orbitofrontal cortex (OFC) (*r* = −0.429, *P* = 0.029, **Figure 5B**). Right OFC activation was significantly related to the inhibitory control (commission error rate) (*r* = 0.439, *P* = 0.028, **Figure 5B**). Left hippocampus activation was significantly associated with both attention bias score (*r* = 0.598, *P* = 0.0001, **Figure 5C**) and attention orienting score (*r* = 0.446, *P* = 0.022, **Figure 5C**). Moreover, left hippocampus activation was negatively correlated with weight percent change (*r* = −0.452, *P* = 0.02, **Figure 5C**). Left amygdala activation was significantly associated with attention orienting score (*r* = 0.410, *P* = 0.038, **Figure 5D**).

## Discussion

To elucidate the multifaceted mechanisms of hypothalamic obesity, we comprehensively performed multiple-dimensional analyses of hypothalamic structural damage, whole-brain response to food images, food-related cognition, food-related psychological characteristics, eating behaviors, and weight gain. Compared with controls, patients with HD showed greater postoperative weight gain, lower neural activation in the left caudate nucleus in response to food images, higher food inattention, lower satiety, and higher restrained eating behavior. Within the HD group, a higher restrained eating behavior was significantly associated with lower activation in the bilateral fusiform gyrus.

Patients with HD showed reduced activation in the left caudate nucleus and increased activation in the occipital gyrus and inferior frontal gyrus in response to food images. This reduced activation in the caudate nucleus, which plays a key role in controlling visual attention and has been linked to attention-deficit hyperactivity disorder (37–39), may explain the lower attention to food observed in the Go/NoGo task.

Moreover, since satiety is closely related to food reward, the present results of lower satiety could be explained by impaired activation of the caudate nucleus, which has a major role in food reward processing (37). The occipital gyrus is well known to be the visual processing center of the brain (38), and the inferior frontal gyrus has been reported to have an important role in visual attention (39). Therefore, it could be concluded that the brain regions with increased activation were associated with processing visual stimuli. In short, these between-group differences suggest that hypothalamic damage could have altered brain function (attention to food images), which could be related to abnormal cognitive and behavioral responses to food and eating.

Patients with hypothalamic damage demonstrated impaired attention to food images and lower satiety. Our results are partly in concordance with previous studies that reported an altered perception of food images (26), increased eating disorder symptoms (7, 9), and less efficient use of executive control processes (12). The lower attention to food could be the major driver of lower satiety (higher hunger) and abnormal eating behavior. Lower attention to eating (i.e., mindless eating) is suggested as one of the major causes of obesity (40, 41). Furthermore, paying attention to food has been proposed to be a better solution for maladaptive eating behaviors (42). These findings suggest that a frequent mindless eating pattern could be a unique pathogenic mechanism of hypothalamic obesity, due to low satiety and low attention to food.

Higher restrained eating behavior was observed in patients with hypothalamic damage. According to previous studies, restrained eating behavior results in binge eating and overeating once food is available (43, 44), which leads to weight gain. A higher restrained eating score has been reported in patients with childhood-onset craniopharyngioma (7, 10, 45). The present study validates these findings and further expands the findings in patients with adult-onset craniopharyngioma.

In patients with hypothalamic damage, brain responses in the fusiform gyrus, OFC, hippocampus, and amygdala correlated with restrained eating, hypothalamic damage, disordered eating attitude, inhibitory control, attention bias, attention orienting, or weight percent change. Higher brain response in the fusiform gyrus was associated with lower restrained eating. This result is consistent with recent reports showing an association between the fusiform gyrus and distorted body image perception among patients with eating disorders (24, 46, 47). The brain response in the OFC was associated with a disordered eating attitude and inhibitory control. The OFC is a well-known brain region related to behavior control (25, 48). The brain response in the hippocampus was associated with weight gain and attention. The hippocampus is well-known to regulate selective attention and energy balance (49, 50). Hippocampal damage may interfere with the inhibition of appetitive and eating behaviors (51). The amygdala is recognized to have an important role in attention and is related to food reward processing (25, 52). These findings provide mechanistic links through which hypothalamic damage could lead to brain network dysfunctions and psychological problems.

The major strength of our study is that it is the first to provide direct evidence to elucidate the mechanism of the link between hypothalamic damage and neural/psychological/behavioral dimensions and health outcomes. Particularly, this study investigated cognitive dysfunctions in patients with craniopharyngioma and endeavored to establish their correlation with psychological and brain dysfunctions. In addition, to the best of our knowledge, this is the only adult-onset craniopharyngioma fMRI study with an adequate control group and a sufficient number of hypothalamic damage patients (compared to a similar previous study with four patients) (26).

Regarding limitations, first, we have used a neuroimaging statistical threshold level (uncorrected *P* < 0.005) that has been recommended by previous studies (53, 54). This may lead to a probability of false positivity. Second, the present study did not have measurements available of resting energy expenditure or circadian rhythm. Third, we only conducted fMRI scans at the baseline level. We did not assess brain responses related to satiety due to difficulties in controlling the internal state in our outpatient setting. Since the hypothalamus is associated with the internal state, further research is suggested to investigate the association between various eating-related phenotypes and brain responses regarding the internal state. Fourth, since hypothalamic obesity occurs in only around half of the patients who have undergone surgical removal of craniopharyngioma, a considerable proportion of patients with HD shared similar clinical features with the control group. A future study focused on more severe HD patients would provide more severe phenotype results. Finally, the present study was an explorative study to discover hypothalamic obesity-related cognitive and neurofunctional mechanisms using various phenotypes. Therefore, due to multiple comparisons, there is a substantial risk of reporting false-positive results.

In conclusion, this study elucidated that hypothalamic damage could lead to dysfunction in brain regions related to attention and food reward processing and result in food inattention, restrained eating behavior, and weight gain. This understanding of the pathological mechanisms could break new grounds on psycho-behavioral therapeutics targeted for hypothalamic obesity patients.

## Data availability statement

The original contributions presented in the study are included in the article/**Supplementary Material**. Further inquiries can be directed to the corresponding authors.

## Ethics statement

The studies involving human participants were reviewed and approved by the Institutional Review Board of Seoul National University Hospital. The patients/participants provided their written informed consent to participate in this study.

## Author contributions

Conception and design: HC, YK and JK. Acquisition of data: ML and M-JP. Analysis and interpretation of data: ML and HC. Drafting of the article: ML and HC. Statistical analysis: ML. Study supervision: KL, JK, YK and HC. All authors contributed to the article and approved the submitted version.
