# Supplementary material for: Obesity mechanism after hypothalamic damage: Cohort analysis of neuroimaging, psychological, cognitive, and clinical phenotyping data
Source: Front Endocrinol (Lausanne). 2023 Mar 28;14:1114409. doi: 10.3389/fendo.2023.1114409 (PMC10086156; doi:10.3389/fendo.2023.1114409)
Supplement: Supplementary file 1 [file Table_1.docx]

Supplementary Materials for

**Obesity mechanism after hypothalamic damage: Cohort analysis of neuroimaging, psychological, cognitive and clinical phenotyping data**

Miwoo Lee, Min-Jung Park, Kyung Hwa Lee, Jung Hee Kim, Hyung Jin Choi and Yong Hwy Kim

*Corresponding author. Email: [kimyh96@snu.ac.kr](mailto:kimyh96@snu.ac.kr), [hjchoi@snu.ac.kr](mailto:hjchoi@snu.ac.kr)

**This PDF file includes:**

Tables S1 to S2

Table S1.

Regions in which brain activation during food image perception was significantly different HD(n=26) and Control(n=28) group

|  | Cluster | Cluster size(k) | x | y | z | Z-value | Region | % | Voxel |
| --- | --- | --- | --- | --- | --- | --- | --- | --- | --- |
| CP> | 1 | 51 | 26 | -66 | 34 | 3.30 | R Superior occipital gyrus | 50.98 | 26 |
|  |  |  |  |  |  |  | R Middle occipital gyrus | 37.25 | 19 |
|  | 2 | 38 | -40 | 30 | 16 | 3.30 | L Inferior frontal gyrus, triangular part | 100 | 38 |
|  | 3 | 36 | 2 | -78 | -28 | 3.20 | L Lobule VII of vermis | 58.33 | 21 |
|  |  |  |  |  |  |  | L Crus II of cerebellar hemisphere | 41.67 | 15 |
| CP< | 1 | 47 | -6 | 2 | 14 | 3.86 | L Caudate nucleus | 38.3 | 18 |

Whole-brain analysis result table. Significance level: uncorrected p<0.005, k>30, presented voxel>5

Table S2.

fMRI cluster coordinate (HO(n=14) vs the others(n=40))

|  | Cluster | Cluster size(k) | x | y | z | Z-value | Region | % | Voxel |
| --- | --- | --- | --- | --- | --- | --- | --- | --- | --- |
| HO> | 1 | 176 | -50 | -26 | 12 | 4.10 | L Superior temporal gyrus | 65.34 | 115 |
|  |  |  |  |  |  |  | L Rolandic operculum | 18.75 | 33 |
|  |  |  |  |  |  |  | L SupraMarginal gyrus | 10.80 | 19 |
|  |  |  |  |  |  |  | L Postcentral gyrus | 4.55 | 8 |
|  | 2 | 113 | -22 | 36 | 68 | 3.88 | L Postcentral gyrus | 92.92 | 105 |
|  | 3 | 124 | 40 | -8 | 42 | 3.82 | R Precentral gyrus | 65.32 | 81 |
|  | 4 | 39 | -54 | -50 | -8 | 3.82 | L Inferior temporal gyrus | 74.36 | 29 |
|  |  |  |  |  |  |  | L Middle temporal gyrus | 25.64 | 10 |
|  | 5 | 81 | -36 | -14 | 8 | 3.52 | L Insula | 45.68 | 37 |
|  |  |  |  |  |  |  | L Lenticular nucleus, Putamen | 13.58 | 11 |
|  | 6 | 33 | 26 | -74 | 12 | 3.32 | R Superior occipital gyrus | 42.42 | 14 |
|  |  |  |  |  |  |  | R Carcarine | 18.18 | 6 |
|  | 7 | 34 | -26 | -84 | 10 | 3.25 | L Middle occipital gyrus | 76.47 | 26 |
|  | 8 | 37 | 48 | -18 | 22 | 3.23 | R Rolandic operculum | 62.16 | 23 |
|  | 9 | 30 | 26 | -66 | 32 | 3.22 | R Superior occipital gyrus | 86.67 | 26 |
|  | 10 | 35 | 22 | -78 | 0 | 3.09 | R Lingual gyrus | 91.43 | 32 |
|  | 11 | 46 | -38 | 22 | 24 | 3.18 | L Inferior frontal gyrus | 100 | 46 |
|  | 12 | 31 | -6 | 8 | 54 | 2.99 | L Supplementary motor area | 100 | 31 |
| HO< | 1 | 30 | 10 | -34 | -14 | 3.34 | R Lobule III of cerebellar  hemisphere | 30 | 9 |
|  | 2 | 50 | 14 | 44 | 4 | 3.27 | R Anterior cingulate cortex | 54 | 27 |
|  |  |  |  |  |  |  | R Superior frontal gyrus | 46 | 23 |

Whole-brain analysis result table. Significance level: uncorrected *p*<0.005, k>30, presented voxel>5
